# Investigating the functions and molecular mechanisms of DTX3L in cancer progression

**DOI:** 10.3389/fonc.2026.1785147

**Published:** 2026-03-10

**Authors:** Haiyu Mu, Mengwen Han, Hejuan Hu, Xilin Xu, Haojian Wang, Feng Zhu, Yongxia Wu, Liangqian Jiang, Yao Chen

**Affiliations:** 1 School of Public Health, Suzhou Vocational Health College, Suzhou, Jiangsu, China; 2 SIPSG Information Technology Company Limited, Suzhou, Jiangsu, China; 3 School of Life Sciences, Jining Medical University, Jining, Shandong, China; 4 Obstetrics, Linyi Peoples’ Hospital, Linyi, Shandong, China; 5 Department of Laboratory Medicine, Linyi Peoples’ Hospital, Linyi, Shandong, China

**Keywords:** cancer, cancer biomarker, DTX3L, E3 ubiquitin ligases, molecular mechanism

## Abstract

In the field of tumor research, the process by which E3 ubiquitin ligases specifically ubiquitinate substrate proteins and promote their degradation has garnered significant attention. DTX3L, an emerging E3 ubiquitin ligase of the Deltex family, is closely associated with the development and progression of various malignant tumors. Through multiple mechanisms such as epigenetic modifications, signal pathway activation, and protein interaction networks, DTX3L precisely regulates key biological processes, including cell proliferation, cycle progression, migration, invasion, and apoptosis. This review systematically summarizes cutting-edge research findings on DTX3L across numerous malignant tumors and analyzes its regulatory mechanisms and functional manifestations in these cancers. From a clinical translation perspective, DTX3L, with its unique substrate selectivity, is becoming a promising cancer therapeutic target. However, therapeutic strategies targeting DTX3L require more in-depth and thorough validation for clinical translation. In summary, DTX3L is expected to serve as a crucial bridge connecting basic research with clinical applications, playing a significant role in cancer precision diagnosis and treatment.

## 1 Introduction

Ubiquitination, a pivotal post-translational modification, has drawn extensive attention across diverse fields, particularly immunology and tumor biology. As the core component of the E1 - E2 - E3 ubiquitination cascade, E3 ubiquitin ligases play a critical role in numerous biological processes, driving a surge in research interest (1). Accumulating evidence shows that diverse E3 ligases (HECT, RING, and RBR types) promote tumorigenesis by modulating proliferation, cell-cycle progression, apoptosis, autophagy, and inflammation (2). Although some E3 ligases function solely as oncogenic drivers or tumor suppressors, others can switch between these roles depending on the cellular context. Unlike E1, E2, or proteasome subunits, E3 ligases exhibit high substrate specificity, making them attractive therapeutic targets that can achieve potent efficacy with minimal off-target toxicity (2). Moreover, because a single E3 ligase can ubiquitinate and degrade multiple oncoproteins, targeting E3 ligases is a more strategic approach than inhibiting an individual driver oncogene.

Among these, DTX3L (Deltex E3 ubiquitin ligase 3L) has gained prominence due to its complex roles in both cancer biology and immune regulation. Recent discoveries have particularly highlighted its significance in mediating resistance to cancer immunotherapy, revealing a critical knowledge gap. For instance, the PARP9-DTX3L heterodimer has been shown to regulate DNA damage repair and interferon signaling. Despite these pivotal findings, a comprehensive synthesis of DTX3L’s functions—spanning from its classical roles in substrate degradation (e.g., p53, EGFR) to its emerging non-canonical, catalysis-independent functions in chromatin remodeling and immune evasion—remains elusive. This review is therefore timely and necessary to integrate these disparate lines of evidence.

This article consolidates the latest research on DTX3L across various malignancies, including its original association with diffuse large B-cell lymphoma and its expanding role in solid tumors such as triple-negative breast cancer, glioma, and pancreatic cancer (3). We delve beyond its regulation of substrate stability to examine its multifaceted interactions in key signaling pathways. Furthermore, we rigorously evaluate DTX3L’s emerging potential not only as a cancer biomarker but as a novel therapeutic target, especially in the context of overcoming treatment resistance.

## 2 Properties of DTX3L

### 2.1 The expression and subcellular localization of DTX3L

The full-length of DTX3L cDNA encodes a 740 amino acid protein, containing two putative nuclear localization signals (PRVRRKL [amino acid: 20-26] and RKHLHQTKFADDFRKRH [amino acid: 462-478]) and a nuclear export signal (LNHQFTKLLI [amino acid: 325-334]) (3, 4), allowing endogenous DTX3L to localize to both the nucleus and cytoplasm. Studies have confirmed endogenous DTX3L in the cytoplasm and nucleus (5, 6), consistent with the Human Protein Atlas data. In cancer drug resistance research, when paclitaxel-treated vemurafenib- resistant SKMEL28 (SKMEL28 - R) cells were studied, DTX3L relocalized from the cytoplasm to the nucleus upon paclitaxel treatment, reducing the cell line’s metastatic ability (7).

**Figure 1:**
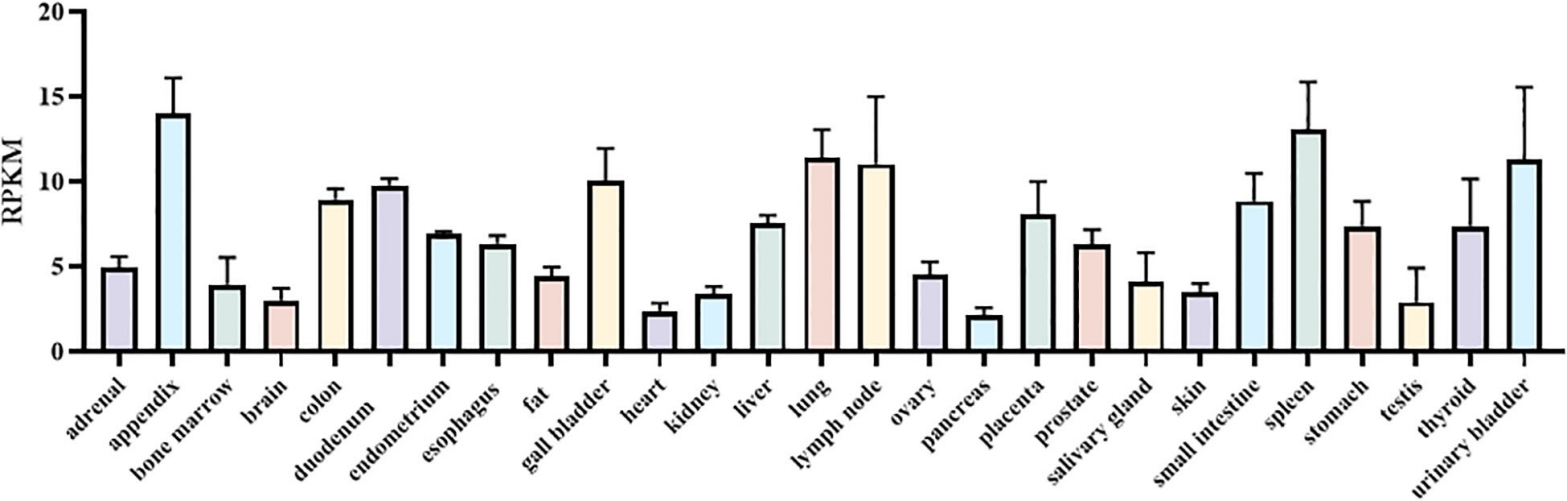
The expression of DTX3L across human tissues. RNA-seq was performed of tissue samples from 95 human individuals representing 27 different tissues in order to determine tissue-specificity of DTX3L. Available online: https://www.ncbi.nlm.nih.gov/gene/151636 (accessed on 1 July 2025).

Using the HPA RNA-seq normal tissues, we detected DTX3L expression across human tissues. DTX3L is highly expressed in multiple tissues, including the appendix, colon, duodenum, gall bladder, liver, lung, lymph node, placenta, small intestine, spleen, stomach, thyroid, urinary bladder (**Figure 1**).

### 2.2 The structure and activities of DTX3L

**Figure 2:**
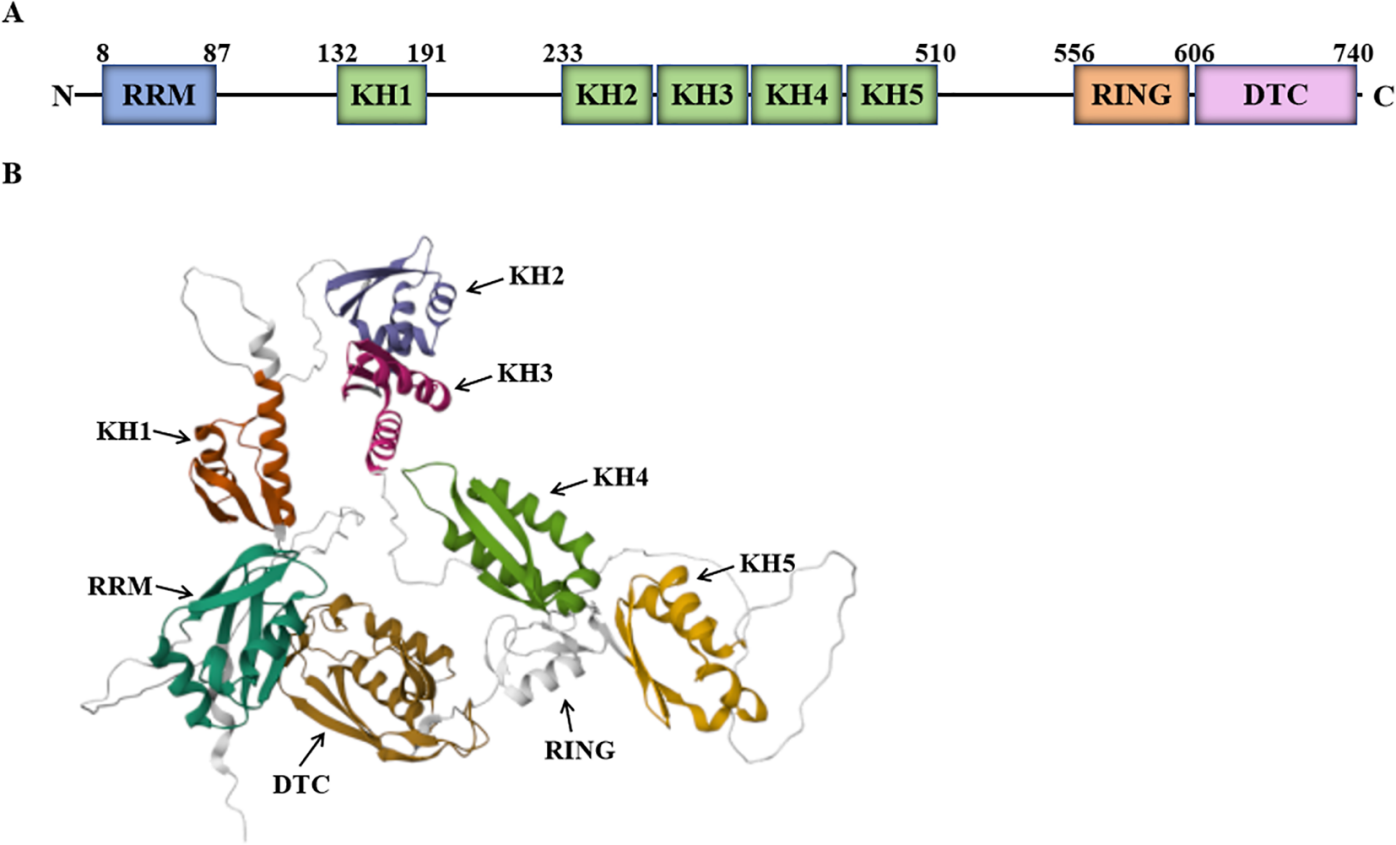
Schematic diagram of DTX3L structure. **(A)** The schematic diagram of DTX3L domain. **(B)** Cartoon representation of DTX3L structure predicted by AlphaFold. Available online: https://alphafold.ebi.ac.uk/entry/Q8TDB6 (accessed on 1 July 2025).

Human DTX3L, composed of 740 amino acids with an estimated molecular weight of 84 kDa, is a multi-domain protein. It contains an RRM domain (RNA recognition motif 1), five KH domains (K homology RNA-binding domain), a RING domain (RING finger domain) and a DTC domain (Deltex C-terminal domain) (**Figure 2**) (8–10).

The RRM domain is located at the N-terminus of the DTX3L protein (amino acid: 8-87). As one of the most abundant protein domains in eukaryotes, the RRM domain, also known as the RNA-binding domain (RBD) or ribonucleoprotein domain (RNP), is highly plastic, enabling high-affinity and specific RNA binding (9–11). In addition to RNA binding, RRM can participate in protein-protein interactions (12).

The KH domain, first identified in hnRNP K, is also called the K homology (KH) RNA-binding domain and binds to single-stranded RNA or single-stranded DNA (9, 10, 13). Single KH domains bind RNA with micromolar affinity (14); the binding affinity of KH domain-containing RBPs is usually enhanced when multiple KH domains are arrayed or partnered with other RNA-binding domains (15). Moreover, individual KH domains within PARP9, PARP14, and DTX3L mediate protein-protein interactions, potentially via mutual dimerization. Consequently, these domains coordinate protein-protein interactions to promote cancer cell survival (8).

The RING domain is found at amino acids 556–611 of the DTX3L structure. Belonging to the HC subclass, the RING finger domain is present in DTX3L protein and similar proteins. The RING domain confers E3 ubiquitin ligase activity by scaffolding the E2~ubiquitin conjugate and allosterically activating the E2 to transfer ubiquitin to a substrate lysine (16, 17), thereby directing protein degradation or functional regulation (10, 18).

The DTC domain is located at the C-terminus of the DTX3L protein (amino acid: 607-739). Since the RING and DTC domains are closely adjacent in the primary sequence, the DTC domain assists RING-mediated ubiquitination. Specifically, the DTC domain catalyzes both ADP-ribosylation of ubiquitin (Ub) and substrate ubiquitination, activities essential for Deltex E3 ligase function. Notably, DTX3L alone transfers ADP-ribose (ADPr) directly to Ub. Together, these domains constitute the minimal catalytic unit required within the DTX family for Ub ADP-ribosylation. During this reaction, the DTC domain binds NAD^+^ while the RING domain recruits the E2~Ub thioester complex; proper spatial arrangement of both domains is essential for catalysis (19, 20). Thus, the DTC domain’s molecular function is likely crucial for DTX3L protein (10, 21).

DTX3L exhibits diverse molecular functions and characteristics. First, it possesses E3 ubiquitin ligase activity, enabling the ubiquitination of multiple tumor related proteins, such as p53, TIRR, Runx2, EGFR, cGAS and LIPG (22–27). This action regulates the stability and function of target proteins. Second, DTX3L can bind to proteins of the STAT family. It forms a functional complex with PARP9, counteracting the phosphorylation status of STAT1 Y701 during tumor development. This precisely modulates the nuclear activities of STAT1α and STAT1β, thereby influencing cellular behavior (28). Furthermore, DTX3L combines with ADP ribosyltransferase PARP9 to participate in DNA damage repair and interferon-mediated antiviral responses (3, 5, 6, 29). DTX3L also monoubiquitinates several histones, including H2A, H2B, H3, and H4 (6, 30). This promotes chromatin remodeling and positively regulates STAT1-dependent interferon stimulated gene transcription, thus modulating STAT1-mediated viral replication control. Overall, DTX3L plays a multidimensional regulatory role in malignant tumor development and is involved in key biological processes of tumor cells, such as proliferation, cell cycle regulation, migration, invasion, apoptosis, chemoresistance and DNA damage repair.

## 3 The role of DTX3L in several cancers

**Figure 3:**
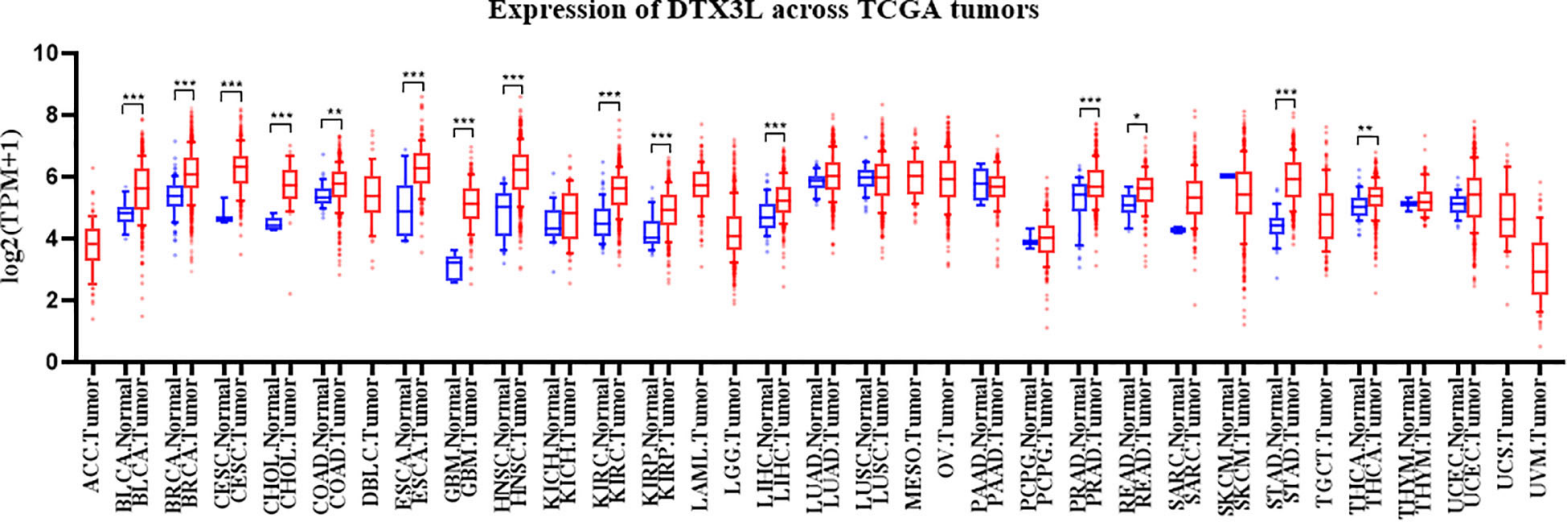
The mRNA levels of DTX3L in tumor tissues and adjacent normal tissues according to the TCGA database. *P < 0.05, **P < 0.001 and ***P < 0.0001.

Recently, it has been found that DTX3L can modulate the growth and development of various cancers through its E3 ubiquitin ligase activity. We used TIMER 2.0 to explore DTX3L expression in pan-cancer tissues and assessed its expression across 33 cancer tissues and adjacent normal tissues (**Figure 3**). Statistically, in 18 cases, including BLCA (bladder urothelial carcinoma), BRCA (breast invasive carcinoma), CESC (cervical squamous cell carcinoma and endocervical adenocarcinoma), CHOL (cholangiocarcinoma), COAD (colon adenocarcinoma), ESCA (esophageal carcinoma), GBM (glioblastoma multiforme), HNSC (head and neck squamous cell carcinoma), KICH (kidney chromophobe), KIRC (kidney renal clear cell carcinoma), KIRP (kidney renal papillary cell carcinoma), LIHC (liver hepatocellular carcinoma), LUAD (lung adenocarcinoma), PRAD (prostate adenocarcinoma), READ (rectum adenocarcinoma), STAD (stomach adenocarcinoma), THCA (thyroid carcinoma) and UCEC (uterine corpus endometrial carcinoma), DTX3L mRNA expression was up - regulated. The increased expression of DTX3L in different cancers is essential for cell proliferation and oncogenesis. Several studies indicate that high DTX3L expression correlates with poor prognoses in various malignancies (31). To fully understand DTX3L’s role in different cancers, researchers need to clarify its regulatory effects on downstream targets.

### 3.1 Cervical cancer

In terms of global female incidence and mortality, cervical cancer ranks as the fourth most common cancer and is the most prevalent malignant gynecological tumor (32). GLOBOCAN 2022 reports an estimated 660,000 new cervical cancer cases and 350,000 related deaths worldwide in 2022 (32). Persistent infection with high-risk human papillomavirus (HPV), especially HPV16 and HPV18, is the primary etiological factor for cervical cancer (33). Additionally, HIV infection, smoking, immune-related factors, and the deregulation of oncogenes and tumor suppressor genes also contribute to its development (34). Early-stage tumors are commonly treated with a combination of radiotherapy, chemotherapy, and surgery. However, advanced-stage patients often have poor prognoses due to tumor metastasis and recurrence (35).

DTX3L is overexpressed in cervical cancer tissues (36, 37). *In vitro* and *in vivo* experiments show that DTX3L knockdown inhibits cervical cancer cell proliferation, migration, invasion and xenograft formation while promoting apoptosis (8, 29, 36). In SiHa and HeLa cells, DTX3L knockdown significantly reduces Snail, Vimentin, and N-cadherin expression and increases E-cadherin expression, indicating that DTX3L may promote tumor migration and invasion. Additionally, DTX3L knockdown downregulates the anti-apoptotic protein Bcl-2 and upregulates the pro-apoptotic proteins P53 and cleaved Caspase-3 (36). Mechanistically, DTX3L silencing markedly reduces the expression of p-4EBP1/4EBP1, p-p70S6K/p70S6K, p-PI3K/PI3K, and p-AKT/AKT, demonstrating its inhibitory effect on the PI3K/AKT/mTOR pathway and suggesting DTX3L may promote cervical cancer progression through this pathway (36). Moreover, in cervical cancer and oral squamous cell carcinoma, the stability of PARP14 is regulated by the PARP9-DTX3L complex. Depleting both PARP9-DTX3L and PARP14 reduces cell line survival and proliferation. Mechanistically, the KH-like domain in PARP9/DTX3L and PARP14 coordinates protein-protein interactions to promote cancer cell survival (8).

DTX3L plays a significant role in DNA damage repair. When cervical cancer cells are exposed to DNA damaging agents, DTX3L and PARP9 are recruited to DNA damage sites to form the PARP9-DTX3L complex. This complex colocalizes with PARP1 and poly(ADP-ribose) (PAR). After DNA damage, PARP9-DTX3L complex physically binds to PARylated proteins, participating in early ubiquitin chain formation. The early ubiquitination at DNA damage sites requires PARP1, PARP9 and DTX3L. Notably, PARP1 activation and the recruitment of the PARP9-DTX3L complex to DNA damage sites are independent of ATM (ATM serine/threonine kinase) and MDC1 (mediator of DNA damage checkpoint 1). Unlike the ATM-MDC1-RNF8 (ring finger protein 8) pathway, the PARP1-dependent PARP9-DTX3L-mediated DNA damage repair mechanism is functionally distinct and non-redundant. It facilitates the early recruitment of 53BP1, RAP80 and BRCA1 to DNA damage sites and regulates their localization through ubiquitination (5). As an E3 ligase, DTX3L enhances tumor cell survival and reduces the efficacy of cytotoxic therapy by monoubiquitinating histone H4K91 (29). Moreover, DTX3L regulates the abundance of the histone H4K20 methyltransferase PR-Set7/Set8. Depleting DTX3L reduces H4K20 monomethylation and dimethylation, selectively modulates 53BP1 foci formation, and increases cellular sensitivity to DNA damage (29).

DTX3L can be combined with various chemotherapeutic drugs for cervical cancer therapy. DTX3L restricts cellular responses to DNA damaging agents like hydroxyurea and doxorubicin. Depleting DTX3L enhances the cytotoxic effects of these drugs in HeLa cells. DTX3L may increase tumor cell survival and reduce the efficacy of cytotoxic therapy through histone H4K91 monoubiquitination (29). Compared to cisplatin alone, DTX3L silencing combined with cisplatin further downregulates Bcl-2 and upregulates cleaved Caspase-3 in HeLa and SiHa cells, significantly promoting cervical cancer cell apoptosis (36).

### 3.2 Melanoma

Cutaneous melanoma, originating from melanocytes at the epidermal-dermal junction of the skin, is a highly aggressive malignant tumor with a higher metastatic potential (38, 39). Its incidence is increasing at a faster rate than that of other cancers. Current therapies include surgical resection, pharmacotherapy, chemotherapy, targeted therapy, and immunotherapy. However, these treatments are influenced by tumor stage and drug side effects (40).

In melanoma, DTX3L is highly expressed in mouse models and human cells. Research indicates that DTX3L levels are significantly higher in RFP/RET transgenic mice and human melanoma cell lines than in benign melanocytes and normal human epidermal melanocytes. Over 80% of melanoma tissues highly express DTX3L, as confirmed by immunohistochemical analysis of human tissues (41). DTX3L was validated as prognostic genes (42). *In-vitro* and *in-vivo* experiments show that DTX3L knockdown reduced melanoma cell migration, invasion, and xenograft tumor formation. Further research shows that in B16F10 and G361 cells, DTX3L silencing significantly reduced p-Fak, p-PI3K, and p-AKT expression, with no significant changes in p-Mek and p-Erk levels. This indicates that DTX3L regulates melanoma invasion and metastasis primarily through the FAK/PI3K/AKT pathway rather than the MEK/ERK pathway (41).

In vemurafenib-resistant SKMEL28 (SKMEL28-R) cells, taxol treatment shifts DTX3L localization from the cytoplasm to the nucleus, reducing the cell line’s metastatic ability (7). This change in DTX3L subcellular localization alters cell morphology and affects migration and invasion by modulating protein expression. Specifically, it downregulates p-FAK and N-cadherin, upregulates p21 and E-cadherin, and reduces SKMEL28-R cell metastasis and invasion (7). These findings highlight DTX3L’s significant role in melanoma metastasis regulation, indicating its potential as a novel biomarker and therapeutic target for metastatic melanoma.

### 3.3 Prostate cancer

Prostatic cancer is one of the common malignant tumors in the male reproductive system (32, 43). According to GLOBOCAN 2022 statistics, the number of new cases of prostate cancer accounted for 7.3% of all new cancer cases globally in 2022 (32). It is projected that by 2025, prostate cancer will account for 30% of all cancer cases among American males. The optimal treatment strategy for prostate cancer involves risk assessment, taking into account factors such as diagnostic staging, histological grading, patient age, overall health status and serum prostate-specific antigen (PSA) levels (32).

DTX3L is a novel oncogenic survival factor in prostate cancer cells (28). It’s overexpressed in prostate cancer patient specimens (22, 28, 30). In prostate cancer cells, DTX3L mediates proliferation, chemoresistance, and survival in a STAT1-dependent manner. It also regulates cell migration via STAT1-dependent and/or STAT3-dependent mechanisms (22, 28). Moreover, DTX3L inhibits the tumor suppressor IRF1 in PCa cells and interacts with the IFNGR complex. Along with PARP9, DTX3L counteracts STAT1 Y701 phosphorylation in prostate cancer cells, modulating the nuclear activity of STAT1α and STAT1β and fine-tuning STAT1 signaling during tumorigenesis (28). Androgen induces the assembly of the AR-DTX3L/PARP9 complex. Olaparib inhibits this androgen-induced AR-DTX3L/PARP9 complex formation (44). ADP-ribosylated AR is recognized by the PARP9/DTX3L heterodimeric complex. The assembly of the AR-PARP9/DTX3L complex helps regulate the transcription of a subset of AR target genes in prostate cancer cells (45).

In prostate cancer, the expression of DTX3L and its binding partner PARP9 is markedly elevated. The genes encoding DTX3L and PARP9 share an IFN-induced bidirectional promoter. Within prostate cancer cells, DTX3L and PARP9 form a stable heterodimeric complex. This complex facilitates DNA repair via the NHEJ pathway (30). Overexpression of DTX3L inhibits HR and induces chromosomal instability after DNA damage by downregulating TIRR (22). Under normal conditions, TIRR forms a complex with 53BP1 and negatively regulates 53BP1. When DNA damage occurs, TIRR is transported to the cytoplasm via XPO1-mediated nuclear export and undergoes DTX3L-mediated ubiquitination and degradation. However, in prostate cancer, DTX3L overexpression enhances the nuclear export and degradation of TIRR. This leads to increased accumulation of 53BP1 at DNA double-strand break (DSB) sites. The interaction of 53BP1 with DSB chromatin favors NHEJ over HR, making cells more sensitive to PARP inhibitors (22). Furthermore, DTX3L expression induces synthetic lethality with PARP inhibitors (olaparib) in prostate cancer cells. Specifically, DTX3L-overexpressing cells exhibited significantly lower olaparib IC50 values and markedly reduced proliferation compared with controls. These findings demonstrate that DTX3L overexpression enhances cellular sensitivity to PARP inhibition. Thus, DTX3L overexpression can serve as a biomarker for PARP inhibitor sensitivity in prostate cancer and other cancers. In summary, targeting DTX3L may enhance the efficacy of chemotherapy or radiotherapy in prostate cancer.

### 3.4 Breast cancer

Breast cancer is the most common malignant tumor in women worldwide and a leading cause of cancer-related deaths in women. According to GLOBOCAN 2022, the incidence of breast cancer accounted for 11.6% of all global cancer cases, and it was responsible for 6.9% of all cancer-related deaths globally in 2022 (32). The most common treatment methods for women with breast cancer include surgery, adjuvant radiotherapy, targeted therapy, immunotherapy, and endocrine (hormonal) therapy (32, 46).

DTX3L knockdown in breast cancer MCF10DCIS cells inhibits growth, migration, invasion, and cancer stem cell (CSC) spheroid formation (27). Specifically, DTX3L inactivation doubles the EpCAM^+^ cell population in MCF10DCIS and downregulates stemness-related genes (IL6, SOX2, OCT4, ABCG2, PROCR, SOX9). Additionally, DTX3L depletion reduces multiple epithelial-mesenchymal transition (EMT) associated gene expressions (KRT14, Vimentin, FOXC2, TWIST, ZEB1, SLUG, N-cadherin, Fibronectin) while upregulating E-cadherin. Moreover, the DTX3L-LIPG-ISG15 axis is essential for *in-vivo* tumor formation in nude mice (27). Mechanistically, LIPG participates in the oncogenic DTX3L-ISG15 signaling axis. In basal-like triple negative breast cancer (TNBC) cells, DTX3L acts as an upstream activator of the LIPG-ISG15 signaling axis and protects LIPG from protein degradation. These *in-vitro* and *in-vivo* findings indicate that DTX3L is crucial for TNBC’s invasive, stem-like, and EMT characteristics, and that the DTX3L-LIPG-ISG15 axis is vital for basal-like TNBC development (27).

DTX3L ubiquitinates USP28, promoting its proteasomal degradation, whereas USP28 counteracts this by deubiquitinating itself and DTX3L. This interplay fine-tunes all major DNA repair pathways, including non-homologous end joining (NHEJ), homologous recombination (HR), single-strand annealing (SSA), and microhomology-mediated end joining (MMEJ). Consequently, DTX3L and USP28 govern DNA repair and cellular viability. DTX3L knockdown induced pronounced G0/G1 phase arrest, concomitantly reducing S and G2 phase populations (47).

All-trans retinoic acid (ATRA) stimulates viral mimicry, interferon responses and antigen presentation in breast cancer cells, offering great potential for individualized and stratified breast cancer therapy. ATRA induces non-conventional viral mimicry, increasing the expression of IRF1 (interferon regulatory factor 1) and DTX3L. IRF1 and DTX3L proteins are involved in ATRA-induced growth inhibition in breast cancer cells. Functional studies show that IRF1 and DTX3L form a negative feedback loop controlling ATRA-dependent growth inhibition in these cells (48).

### 3.5 Glioma

Glioma is the most common malignant tumor of the central nervous system, with glioblastoma multiforme (GBM) being the most frequent type, accounting for 57.3% of all gliomas (49). It is characterized by diffuse invasive growth and low survival rates. Despite advancements in treatment approaches, such as surgical resection combined with temozolomide (TMZ) based radiotherapy and chemotherapy, the prognosis remains poor. Only 10% of patients survive 5 years after diagnosis, with a median survival period of just 15 months (49, 50). Thus, exploring the molecular mechanisms underlying glioma progression is crucial for identifying potential diagnostic and therapeutic targets.

DTX3L protein expression is significantly higher in glioma tissues than in normal brain tissues. DTX3L expression increases with higher tumor malignancy and correlates positively with Ki-67 expression. Survival analysis shows that DTX3L upregulation is significantly associated with shorter overall patient survival. Experimentally, DTX3L silencing inhibits glioma cell proliferation, migration, and invasion, and induces apoptosis. Further research shows that DTX3L silencing upregulates E-cadherin and downregulates Vimentin, which partly explains its inhibitory effect on cell migration and invasion (51).

Temozolomide (TMZ) is a primary clinical treatment for glioma patients and its efficacy is of significant interest. Research has revealed that DTX3L silencing can enhance the cytotoxic effect of TMZ in U87MG cells. Compared to the groups treated with DTX3L knockdown or TMZ alone, the co-treatment group exhibited increased levels of cleaved caspase-3 and cleaved PARP, as well as a higher proportion of apoptosis cells. This indicates that DTX3L plays a crucial role in TMZ-induced glioma cell apoptosis and that DTX3L knockdown increases glioma sensitivity to TMZ (51).

### 3.6 Pancreatic cancer

Pancreatic cancer ranks among the most deadly human malignancies. According to GLOBOCAN 2022, pancreatic cancer was responsible for 2.56% of global cancer cases and 4.81% of cancer related deaths in 2022 (32). The incidence and mortality of pancreatic cancer are attributed to the interplay of environmental and genetic factors, as well as established risk factors such as smoking, obesity, diabetes, and alcohol abuse (32). Therefore, in-depth research on pancreatic cancer pathophysiology and the development of early diagnostic tools and effective therapies are of utmost importance for improving its prognosis.

DTX3L expression is markedly elevated in pancreatic cancer tissues compared to adjacent non-cancerous tissues. Patients with high DTX3L expression have a significantly lower survival expectancy than those with low expression, and DTX3L expression levels are negatively correlated with patient survival rates. Kaplan-Meier survival analysis shows that high DTX3L expression in tumor tissues is closely associated with poor prognoses in pancreatic cancer patients (23, 24). DTX3L downregulation inhibits cell proliferation, while its upregulation promotes proliferation. In addition, DTX3L enhances cell invasion and migration abilities. Mechanistically, DTX3L binds to EGFR, interfering with its ubiquitination and stabilizing EGFR. The subsequent upregulation of EGFR activates the FAK/PI3K/AKT pathway, driving pancreatic cancer progression. Notably, EGF, an activator of the FAK/PI3K/AKT pathway, can reverse the tumor suppressor effects of DTX3L knockout (24). EGFR binding and activation of the FAK/PI3K/AKT pathway promote tumor progression and affect pancreatic cancer cell sensitivity to chemotherapy and radiotherapy. Thus, DTX3L is a potential therapeutic target for pancreatic cancer.

Pancreatic Cancer cells with DTX3L knockdown show significantly enhanced sensitivity to gemcitabine or FOLFIRINOX, whereas DTX3L overexpression reduces chemotherapeutic drug sensitivity (24). DTX3L knockout enhances radiotherapy efficacy in subcutaneous tumor mouse models and promotes cytokine production in tumor tissues. This indicates that targeting the DTX3L-cGAS axis may be a promising strategy for improving pancreatic cancer radiotherapy outcomes (23).

### 3.7 Esophageal squamous cell carcinoma

Esophageal cancer ranks as the 11th most commonly diagnosed cancer and the 7th leading cause of cancer related mortality globally (32). GLOBOCAN 2022 reports show it accounts for 2.56% of global cancer cases and 4.59% of cancer related deaths (32). Esophageal squamous cell carcinoma (ESCC) is the predominant histological subtype of esophageal cancer in Asian populations (52). Smoking and alcohol consumption are the primary risk factors for ESCC. Consequently, there is an urgent need to develop strategies for treating ESCC at early or precancerous stages.

DTX3L is overexpressed in ESCC tissues compared to normal tissues. DTX3L knockdown significantly inhibits ESCC cell migration and reduces Eca-109 and TE-1 cell proliferation. *In-vivo* experiments show that DTX3L knockdown reduces tumor size and decreases M2 macrophage infiltration, especially pro-tumoral M2 macrophages (53). Tumor associated macrophages (TAMs) mainly exhibit two phenotypes: M1 (anti-tumoral) and M2 (pro-tumoral) (54). Identifying factors that regulate M2 macrophage polarization is crucial for inhibiting TAM driven tumor progression (55). *In-vitro* and *in-vivo* data indicate that DTX3L accelerates ESCC cell malignancy and promotes TAM polarization towards the M2 phenotype. TAMs serve as central hubs of the immunosuppressive network. Together with regulatory T cells (Tregs), myeloid-derived suppressor cells (MDSCs), immunosuppressive erythroid progenitor cells (EPCs), and tumor-associated neutrophils (TANs), they cooperatively establish and maintain an immunosuppressive tumor microenvironment, thereby abrogating effective antitumor immunity (56, 57). Thus, increased DTX3L expression during ESCC progression may facilitate an immunosuppressive tumor microenvironment (TME).

### 3.8 Diffuse large B-cell lymphomas

Diffuse large B-cell lymphomas (DLBCL) is the most common lymphoid malignancy in adults and accounts for approximately 40% of non-Hodgkin lymphomas (NHLs) (32). The first-line treatment for DLBCL typically involves chemotherapy immunotherapy, with a standard regimen combining rituximab, cyclophosphamide, doxorubicin, vincristine and prednisone (46). However, chemoresistance in DLBCL cells is common, leading to high relapse rates and making it a particularly challenging disease to treat.

DTX3L is overexpressed in primary DLBCL. DTX3L reaches the highest expression level in host response (HR) tumors, which are lymphomas with rapid host inflammatory responses (4). These HR DLBCLs feature significant immune and inflammatory infiltration, and IFN-γ likely regulates DTX3L expression. IFN-γ can activate DTX3L promoters and induces DTX3L expression in DLBCL cell lines. Notably, IFN-γ induced DTX3L protein is mainly expressed by tumor cells, highlighting dynamic interactions between HR tumor cells and their microenvironment (4).

EZH2 gain-of-function mutation (EZH2^GOF^) is present in DLBCL cells. EZH2^GOF^ DLBCL cells overexpress DTX3L (58). In DLBCL cells, DTX3L activity can protect cells from DNA damage. The selective inhibition of HDAC1 and HDAC2 can increase H4K91 acetylation, thereby inhibiting DTX3L-mediated H4K91 monoubiquitination and impairing 53BP1 recruitment mediated DNA double-strand break (DSB) repair. This process triggers DNA damage in lymphoma cells and overcomes DTX3L-mediated chemoresistance, thereby re-sensitizing refractory EZH2^GOF^ DLBCL cells to doxorubicin treatment (58).

### 3.9 Hepatocellular carcinoma

Hepatocellular carcinoma (HCC) is the sixth most commonly diagnosed cancer and the third leading cause of cancer related mortality worldwide (32). According to GLOBOCAN 2022, the global incidence rate of hepatocellular carcinoma is 4.33%, and the mortality rate is 7.8% (32). The development of liver cancer is closely associated with risk factors such as liver cirrhosis caused by hepatitis B virus (HBV) or hepatitis C virus (HCV) infection, excessive alcohol consumption, exposure to aflatoxin B1, and metabolic syndrome (59). For localized HCC, curative-intent surgical resection, ablation therapy, or transarterial chemoembolization (TACE) can be used. However, in advanced HCC, these therapies can only modestly prolong survival, and combination therapies might lead to significant side effects (60).

DTX3L is highly expressed in hepatocellular carcinoma tissues. CircEYA3 upregulates DTX3L expression at both the mRNA and protein levels. The mechanism involves circEYA3 interacting with IGF2BP2, thereby upregulating DTX3L mRNA expression, repairing radiation induced DNA damage, and enhancing HCC radioresistance (61). As a circular RNA (circRNA), circEYA3 can participate in cellular processes in various ways, such as altering protein interactions, sequestering proteins, recruiting proteins to chromatin, and forming circRNA-protein-mRNA ternary complexes. IGF2BP2, a key m6A reader, can regulate post-transcriptional gene expression and is involved in various tumor behaviors, such as tumor growth, metastasis, angiogenesis, aerobic glycolysis, immune microenvironment, and drug resistance (62–65). The interplay between m6A methylation and tumor metabolism is intricate: metabolic stress reshapes the m6A landscape, while aberrant m6A deposition modulates core signaling axes (mTOR, MAPK, PI3K-Akt, AMPK, Wnt and Hedgehog) that drive metabolic reprogramming (66). The formation of the circEYA3-IGF2BP2-DTX3L mRNA ternary complex increases HCC radioresistance (61).

### 3.10 Osteosarcoma

Osteosarcoma is a common and highly aggressive malignant bone tumor that primarily affects children and adolescents, and is also a leading cause of cancer related mortality in adolescents (67, 68). The standard treatment for osteosarcoma involves surgery combined with adjuvant chemotherapy. However, the therapeutic outcomes are far from satisfactory, and the long-term toxic side effects and chemoresistance significant challenges to patients’ quality of life (69). Thus, there is an urgent need for novel therapeutic approaches to improve the prognosis and quality of life for osteosarcoma patients.

DTX3L knockdown significantly promotes apoptosis in U2OS cells. Further studies have shown that this promotion is particularly evident when U2OS cells are treated with Dox or γ-ray irradiation. DTX3L depletion enhances the cellular response to DNA damage, leading to increased p53 levels after DNA damage. This, in turn, upregulates the cell cycle inhibitor p21 and the pro-apoptotic gene BAX, ultimately promoting cell apoptosis. This indicates that DTX3L plays a significant role in regulating cell apoptosis (25).

P53, as the master transcriptional regulator and effector of the DNA damage response (DDR), is partially localized to DNA damage sites through interaction with PARP1 (70, 71). In the early stages of the DNA damage response, p53 is recruited to DNA damage sites modified by PARP1. Meanwhile, PARP1 induced PARylation can serve as a docking site for the PARP9/DTX3L complex after DNA damage. When DTX3L approaches p53, it can polyubiquitinate p53 and target it for proteasomal degradation, thereby regulating the stability and function of p53 (25).

### 3.11 Multiple myeloma

Multiple myeloma (MM) is an incurable hematological malignancy that typically originates from malignant proliferation of plasma cells and is primarily characterized by skeletal deformities, renal impairment, anemia, and hypercalcemia (72, 73). It is the second most common hematologic tumor, with primary treatment methods including targeted therapy, chemotherapy, radiotherapy and stem cell transplantation (74). Despite significant advances in MM treatment, the therapeutic outcomes remain unsatisfactory. Thus, identifying effective therapeutic targets for MM is imperative.

DTX3L expression progressively increases during myeloma cell proliferation, and DTX3L knockout suppresses this proliferation. Following DTX3L knockout, PCNA expression is substantially downregulated, cell cycle arrest occurs in the G1 phase, and the expression of CDK2 and cyclin E decreases. This suggests DTX3L may promote myeloma cell proliferation by regulating the cell cycle (75).

DTX3L may also be involved in cell adhesion regulation. DTX3L knockout results in a significant reduction in cell adhesion (75). Cell adhesion mediated drug resistance (CAM-DR) is one of the major obstacles in myeloma treatment, as it impedes chemotherapy-induced tumor cell apoptosis (76, 77). DTX3L knockout reduces CAM-DR and promotes myeloma cell apoptosis, with a significant increase in cleaved caspase-3 and cleaved PARP expression in the presence of chemotherapy. After downregulating the expression of DTX3L, p-AKT levels decrease, indicating that DTX3L is regulated by the FAK signaling pathway and exerts its effects through AKT in myeloma cells. Overall, DTX3L plays a key role in mediating CAM-DR in MM cells by regulating cell cycle progression and cell adhesion via the FAK signaling pathway (75).

## 4 DTX3L in cancer-associated complications

Cancer cachexia is a complex metabolic syndrome caused by malignant tumors, primarily characterized by systemic metabolic disturbances in patients, progressive muscle and fat wasting, weight loss, and progressive failure of systemic organs, and cannot be fully reversed by conventional nutritional support (78–80). In advanced cancer patients, the incidence of cancer cachexia is as high as 60%-80%, and approximately 20% of cancer patients die directly from cancer cachexia (80). Cancer cachexia not only weakens the effectiveness of chemotherapy and radiotherapy, shortens patient survival time, but also significantly impairs patients’ quality of life. Currently, there is a lack of effective clinical treatments. Therefore, accurate clinical diagnosis and effective interventions are of great significance for the long-term survival of advanced cancer patients.

L-carnitine can alleviate cancer cachexia related skeletal muscle fibrosis and maintain skeletal muscle function. Further studies have shown that L-carnitine regulates Runx2 ubiquitination via DTX3L, promoting Runx2 degradation and leading to a decrease in COL1A1, thereby alleviating skeletal muscle fibrosis (SMF) caused by cancer cachexia (26). SMF, a marker of muscular dystrophy and severe muscle injury (81), can be relieved by L-carnitine in cancer.

Runx2, a transcription factor and osteogenic marker, is a promising target for malignant tumor therapy (82, 83). DTX3L can regulate Runx2 ubiquitination and promote its degradation. Studies have shown that Runx2 can directly regulate COL1A1 mRNA levels (84). COL1A1, a key structural component of the extracellular matrix (ECM) and a major component of SMF, can be downregulated to alleviate skeletal muscle degeneration, thus reducing muscle atrophy and strength loss caused by cancer cachexia (85). In cancer cachexia models, L-carnitine reduces fibrosis via the DTX3L-Runx2-COL1A1 axis, indicating DTX3L’s significant role in tumor cachexia.

## 5 Discussion

**Figure 4:**
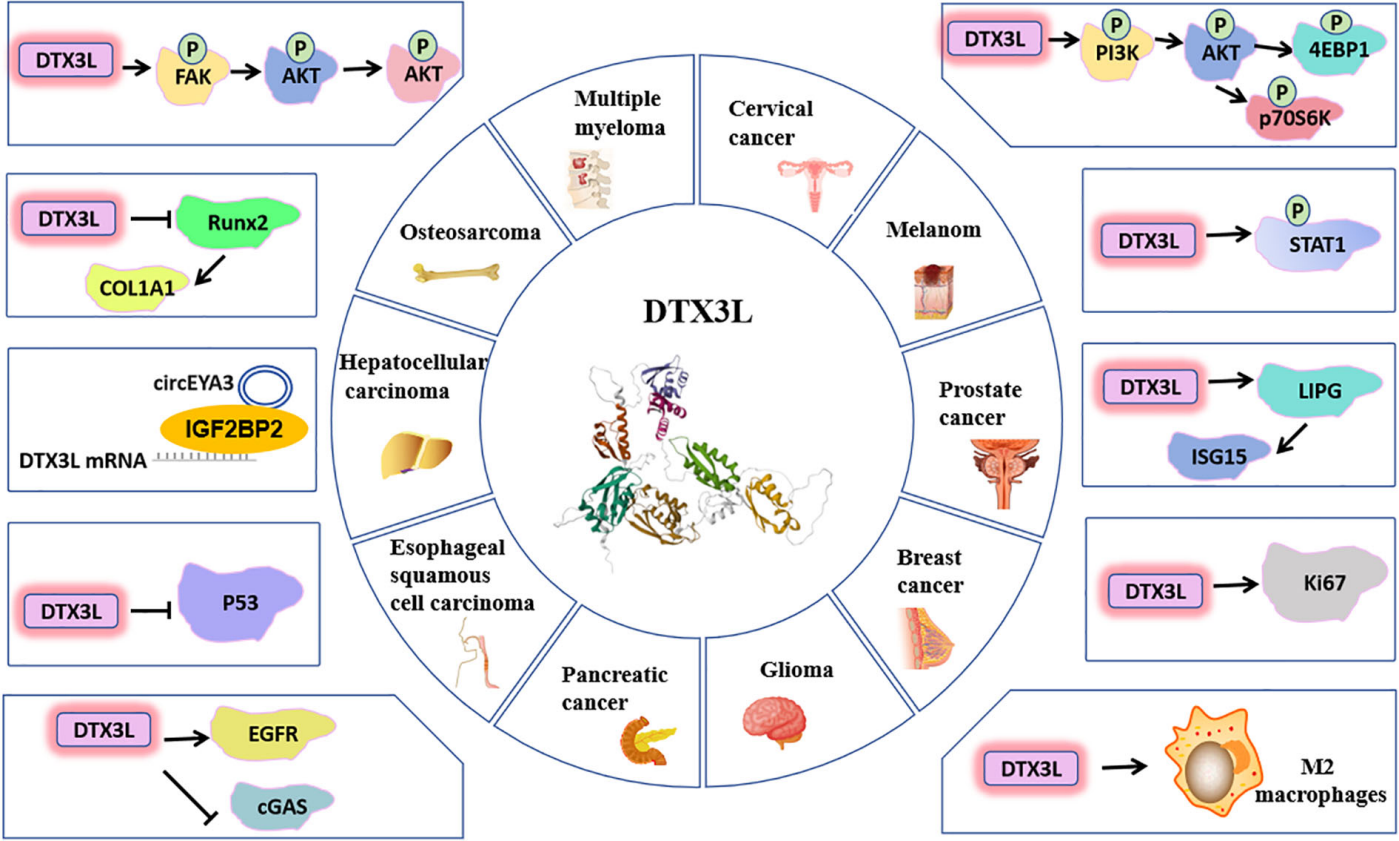
Signaling pathways of DTX3L protein regulating the growth and development of various cancers.

A growing body of evidence indicates that DTX3L as a multifaceted contributor to tumorigenesis, regulating core oncogenic processes including cell proliferation, cycle progression, invasion, metastasis, apoptosis, drug resistance, and DNA-damage repair in a wide range of cancers (**Figure 4**). This central involvement in malignancy underscores its emergence as a promising therapeutic target, with selective inhibition actively explored as a novel antineoplastic strategy. Critically, DTX3L expression levels show significant correlation with key clinical features; its upregulation is consistently associated with poorer prognosis, such as shorter overall survival and higher risk of disease progression in cancers like glioma and pancreatic cancer. These associations highlight its dual potential as both a prognostic biomarker and a therapeutic vulnerability. Furthermore, DTX3L plays a direct role in mediating resistance to chemotherapeutic agents (e.g., hydroxyurea, doxorubicin, cisplatin), suggesting that targeting it could reverse multidrug resistance and resensitize cancer cells to treatment.

Despite its clear oncogenic relevance, the clinical translation of DTX3L targeting faces several hurdles. A primary challenge is the incomplete understanding of its functional heterogeneity across different cancer contexts, which complicates the development of unified targeting strategies. The safety and efficacy profiles of DTX3L inhibition require rigorous validation through comprehensive preclinical studies and subsequent large-scale clinical trials. Future research should prioritize several key directions: (1) elucidating the context-dependent molecular mechanisms of DTX3L to decipher its functional diversity; (2) developing and optimizing specific DTX3L inhibitors or modulators; and (3) investigating synergistic treatment paradigms that combine DTX3L-targeted approaches with conventional chemotherapy, radiotherapy, or immunotherapy to improve clinical outcomes. Addressing these areas will be crucial for harnessing the full diagnostic and therapeutic potential of DTX3L in precision oncology.
